# Assessing Binocular Interaction in Amblyopia and Its Clinical Feasibility

**DOI:** 10.1371/journal.pone.0100156

**Published:** 2014-06-24

**Authors:** MiYoung Kwon, Zhong-Lin Lu, Alexandra Miller, Melanie Kazlas, David G. Hunter, Peter J. Bex

**Affiliations:** 1 Schepens Eye Research Institute/Massachusetts Eye and Ear Infirmary, Boston, Massachusetts, United States of America; 2 Department of Ophthalmology, Harvard Medical School, Boston, Massachusetts, United States of America; 3 Department of Psychology, Ohio State University, Columbus, Ohio, United States of America; 4 Department of Ophthalmology, Boston Children's Hospital, Boston, Massachusetts, United States of America; University College London, United Kingdom

## Abstract

**Purpose:**

To measure binocular interaction in amblyopes using a rapid and patient-friendly computer-based method, and to test the feasibility of the assessment in the clinic.

**Methods:**

Binocular interaction was assessed in subjects with strabismic amblyopia (n = 7), anisometropic amblyopia (n = 6), strabismus without amblyopia (n = 15) and normal vision (n = 40). Binocular interaction was measured with a dichoptic phase matching task in which subjects matched the position of a binocular probe to the cyclopean perceived phase of a dichoptic pair of gratings whose contrast ratios were systematically varied. The resulting effective contrast ratio of the weak eye was taken as an indicator of interocular imbalance. Testing was performed in an ophthalmology clinic under 8 mins. We examined the relationships between our binocular interaction measure and standard clinical measures indicating abnormal binocularity such as interocular acuity difference and stereoacuity. The test-retest reliability of the testing method was also evaluated.

**Results:**

Compared to normally-sighted controls, amblyopes exhibited significantly reduced effective contrast (∼20%) of the weak eye, suggesting a higher contrast requirement for the amblyopic eye compared to the fellow eye. We found that the effective contrast ratio of the weak eye covaried with standard clincal measures of binocular vision. Our results showed that there was a high correlation between the 1^st^ and 2^nd^ measurements (*r* = 0.94, *p*<0.001) but without any significant bias between the two.

**Conclusions:**

Our findings demonstrate that abnormal binocular interaction can be reliably captured by measuring the effective contrast ratio of the weak eye and quantitative assessment of binocular interaction is a quick and simple test that can be performed in the clinic. We believe that reliable and timely assessment of deficits in a binocular interaction may improve detection and treatment of amblyopia.

## Introduction

Amblyopia is an optically uncorrectable loss of vision, usually in one eye, without any known pathology [1]. It is the most common cause of monocular visual loss in children, and affects approximately 3–5% of the population [2]. While amblyopia is associated with a wide range of monocular and binocular visual deficits that include reduced visual acuity [3], loss of contrast sensitivity [4], spatial distortion [1], impaired contour integration [5], abnormal binocular interaction [6], [7], abnormal motion perception [8] and visual-motor deficits [9]–[13], visual acuity remains the main clinical measure for diagnosis and treatment outcomes. Despite the success of penalization and occlusion therapies in improving monocular acuity in the amblyopic eye, the monocular treatment approach has been challenged by high occurrences in residual and recurrent amblyopia [14], hinting that amblyopic vision cannot be fully characterized by a single visual acuity measure.

Evidence has accumulated for a critical role of abnormal binocular visual experience in the residual deficits and the recurrence of amblyopia. Recent population studies have shown that abnormal binocular visual experience, represented by interocular differences in refractive error and poor stereoacuity are good predictors of residual amblyopic deficits [14]–[16]. Furthermore, psychophysical studies have shown the existence of binocular summation in amblyopia under some conditions, at both threshold [17], [18] and suprathreshold levels [7], [19]–[23]. Such interactions are best revealed by compensating for the contrast sensitivity deficit of the amblyopic eye, or equating the effective contrast of the two eyes. Moreover, prolonged exposure to binocularly balanced stimuli has been shown to improve both monocular and binocular vision [24]–[27]. While it has been long believed that amblyopic vision lacks excitatory binocular connections such as binocular summation [28]–[30] and the existence of remaining binocular interactions mostly involve inhibitory mechanisms [31], the recent findings imply that it is possible to restore normal binocularity by addressing the imbalance in monocular signals. These findings, therefore, support a beneficial role of assessing interocular imbalance in detecting and treating amblyopia as well as estimating a patient's prognosis. Several paradigms have been developed to quantitatively assess the interocular imbalance in either normal or amblyopic vision, which includes binocular rivalry [32], dichoptic global motion coherence [33] or orientation [19], binocular phase [7], [34], [35] or/and contrast combination [20], [36]. Furthermore, Black et al. [37] and Li et al. [23] have recently developed a quick and compact version of the test instrument using a dichoptic global motion coherence task. By addressing inefficiency of psychophysical assessments, such as long testing times and the employment of cumbersome apparatus [19], they improved the clinical utility of such assessment. These foregoing studies have successfully demonstrated the efficacy of the rapid and convenient test instrument in assessing binocular interaction in amblyopic individuals and its potential use in the clinic. However, whether such assessment can be indeed carried out as a part of routine clinical assessments still remains to be answered.

Thus, the primary goal of the present study is to examine whether it is feasible to assess binocular interaction in the clinic as a part of routine clinical assessments. In order to assess binocular interaction, we adopted a suprathreshold binocular summation (binocular phase combination) paradigm developed by Ding & Sperling [34]. In this paradigm, two suprathreshold sine-wave gratings of differing contrasts and spatial phases are presented to the left and right eyes of the observer. The perceived phase of the binocularly combined percept is measured as a function of the interocular contrast ratio. Because the perceived phase of the cyclopean grating is determined by the relative strengths of the component sine-wave gratings at the stage of binocular combination, the contribution of each eye to the combined percept can be inferred. This paradigm has been successfully implemented to test binocular interaction in individuals with amblyopia by Huang et al. [7], [20] and by Ding et al. [34], [35]. In the current study, we choose this paradigm because it allows us to examine binocular combination of contrast signals independently of higher-level global motion processing as in the dichoptic global motion coherence task, which may show deficits in both amblyopic and fellow eyes in some cases. [38]

Importantly, our method differs from the original binocular phase combination task in two ways: 1) It requires only a small number of trials to complete the task, taking less than 8 mins of total testing time; 2) It adopts a more user-friendly dichoptic presentation method using stereo-shutter glasses through which subjects receive different images in the two eyes without any need to continually adjust the alignment of the two images, as is the case for stereoscopic mirrors. In order to test the feasibility of the assessment in the clinic, we conducted the assessment in a local ophthalmology clinic during patients' routine visit. Furthermore, an attempt was made to carry out the assessment procedure in a more natural viewing setting in which subjects' binocular fusion was not ensured either by correcting their ocular deviation or a method of binocular alignment procedure. This approach was taken because we aimed to assess the clinical value of binocular interaction assessment when visual acuity alone is available. Next, the efficacy of the method for estimating the interocular imbalance in amblyopia was tested by examining 1) whether subjects with amblyopia exhibit significantly reduced effective contrast ratio (i.e., an indicator of interocular imbalance) compared to subjects with normal vision or strabismus without amblyopia as demonstrated in earlier studies [7], [20], [21], [34], [35]; 2) whether our effective contrast ratio covaries with standard clincal measures indicating abnormal binocular vision such as stereoacuity and interocular acuity difference (IAD); 3) whether our assessment method yields reliable measurements over time.

Our results suggest that, with its short testing time and minimum intervention in testing procedure, assessing binocular interaction as a part of routine clinical assessment is feasible. Incorporating this quantitative measure of binocular function as a part of routine clinical assessment may allow clinicians to more accurately assess individual patients' outcomes and prognosis in addition to standard visual acuity.

## Methods

### Participants

The study design included four groups: (1) patients with strabismic amblyopia, (2) those with anisometropic amblyopia, (3) those with strabismus but without amblyopia, and (4) normally sighted individuals. A complete clinical examination (see below) was performed by clinicians at the Boston Children's Hospital pediatric ophthalmology unit (Boston, USA) and only those who met the following criteria were included: Strabismic amblyopia was defined as a 2-line or greater interocular difference (≥.2 logMAR units) in best-corrected visual acuity with angular deviation between eyes of 5 to 50 prism diopters. Anisometropic amblyopia was defined as a 2-line or greater interocular difference (≥.2 logMAR units) in best-corrected visual acuity with phoria less than or equal to 4 prism diopters. Strabismus without amblyopia was defined as less than a 2-line difference (<.2 logMAR units) in best-corrected interocular visual acuity with angular deviation of 5 to 50 prism diopters. (Note that the onset age of strabismus was around 4 or 5 years for strabismus without amblyopia subjects. Four of strabismus without amblyopia (n = 15) went through patching therapy and another four had received eye muscle surgery in their early childhood.) Normal vision was defined as best-corrected visual acuity better than 0.0 logMAR or uncorrected visual acuity better than 0.33 logMAR for both eyes without any latent or manifest ocular deviation. Subjects with any known cognitive or neurological impairments were excluded from the study.

The experiments were undertaken with the understanding and written informed consent of subjects or the parents (or legal guardian) of subjects aged <18 yrs, in accordance with procedures and protocol approved by the institutional review board of Children's Hospital Boston and complying with the Declaration of Helsinki. Enrolled patients underwent complete clinical examination, including best corrected visual acuity (Snellen charts), cycloplegic refractive error, stereopsis (Titmus Fly SO-001 StereoTest), ocular motility, and binocular fusion test (a Worth 4 dot test) and cover test at near and distance fixation. The angle of any heterotropia or heterophoria was measured by prism-and-cover test at near and distance fixation. In this study, we only reported the measurements made at near fixation, which is more relevant to the 57 cm viewing distance in our experiment. Only children aged 5 years and older participated in our study and they all were able to perform letter acuity assessment. The mean and median age, visual acuity, angular eye deviation, stereoacuity and the gender ratio of subjects in each group are provided in Table 1. All subjects were tested with their best-corrected vision in the psychophysical task (binocular interaction) except for several normally-sighted subjects whose uncorrected visual acuities were between 0.0 and 0.33 logMAR. Hereafter we term the amblyopic eye and fellow eye as the weak eye and strong eye respectively. The strong eye was determined by clinical binocular function tests (for strabismus and amblyopia) or finger pointing task, a variant of the Porta test (for normal).

**Table 1:** Subject characteristics.

			Strabismic amblyopia	Anisometropic amblyopia	Strabismus	Normal
			(N = 7)	(N = 6)	(N = 15)	(N = 40)
**Age (yrs)**	mean (±SD)		22.3 (±19.6)	7 (±1.3)	21.5 (±20.91)	18.7 (±10.7)
	min:median:max		6:8:51	6:6.5:9	5:10:69	5:16:43
**Gender**	ratio (female:male)		2:5	3:3	10:5	19:21
**Visual Acuity**	mean (±SD)	weak eye	0.28 (±0.16)	0.39 (±0.20)	0.06 (±0.08)	0.03 (±0.11)
		strong eye	0.01 (±0.09)	0.03 (±0.08)	0.03 (±0.07)	−0.02 (±0.11)
**(logMAR)**	min:median:max	weak eye	0.14:0.2:0.54	0.12:0.40:0.62	−0.08:0.04:0.17	−0.12:0:0.32
		strong eye	−0.12: −0.02:0.18	−0.12:0:0.09	−0.10:0.02:0.17	−0.22:0:0.32
**Angular**	mean (±SD)		13.6 (±10.1)		18.6 (±14.4)	
**Deviation**	min:median:max		6:10:35	Neither	5:12:50	Neither
**(prism diopter)**			2 XT, 1 INT XT,	latent	7 ET, 1AC ET,	manifest
	type		3 ET, 1 INT ET	manifest nor	3 XT, 4 INT XT	nor latent
				deviation		deviation
	fixational eye		4 FU, 1 LE, 2 RE		10 FU, 2 ALT,	
					2 LE, 1 RE	
**Stereoacuity**	mean (±SD)		492.8 (±396.2)	266.7 (±336.9)	237.3 (±343.9)	47.7 (±18.5)
**(arcsec)**	min:median:max		50:400:900	40:90:900	40:100:900	40:40:100

Note that stereoacuity 900″ is a surrogate for zero stereoacuity for the purpose of computation. SD: Standard deviation, ET: Esotropia, XT: Exotropia, INT: Intermittent, AC: Accommodative, ALT: Alternator, FU: Fuses, Δ: Prism diopter, OD: Right eye, OS: Left eye. Also note that the reported information about ocular deviation and fixational eye are those made at near fixation (see Figs S1 and S2 for the data from all individual subjects).

### Stimuli and Apparatus

**Figure 1:**
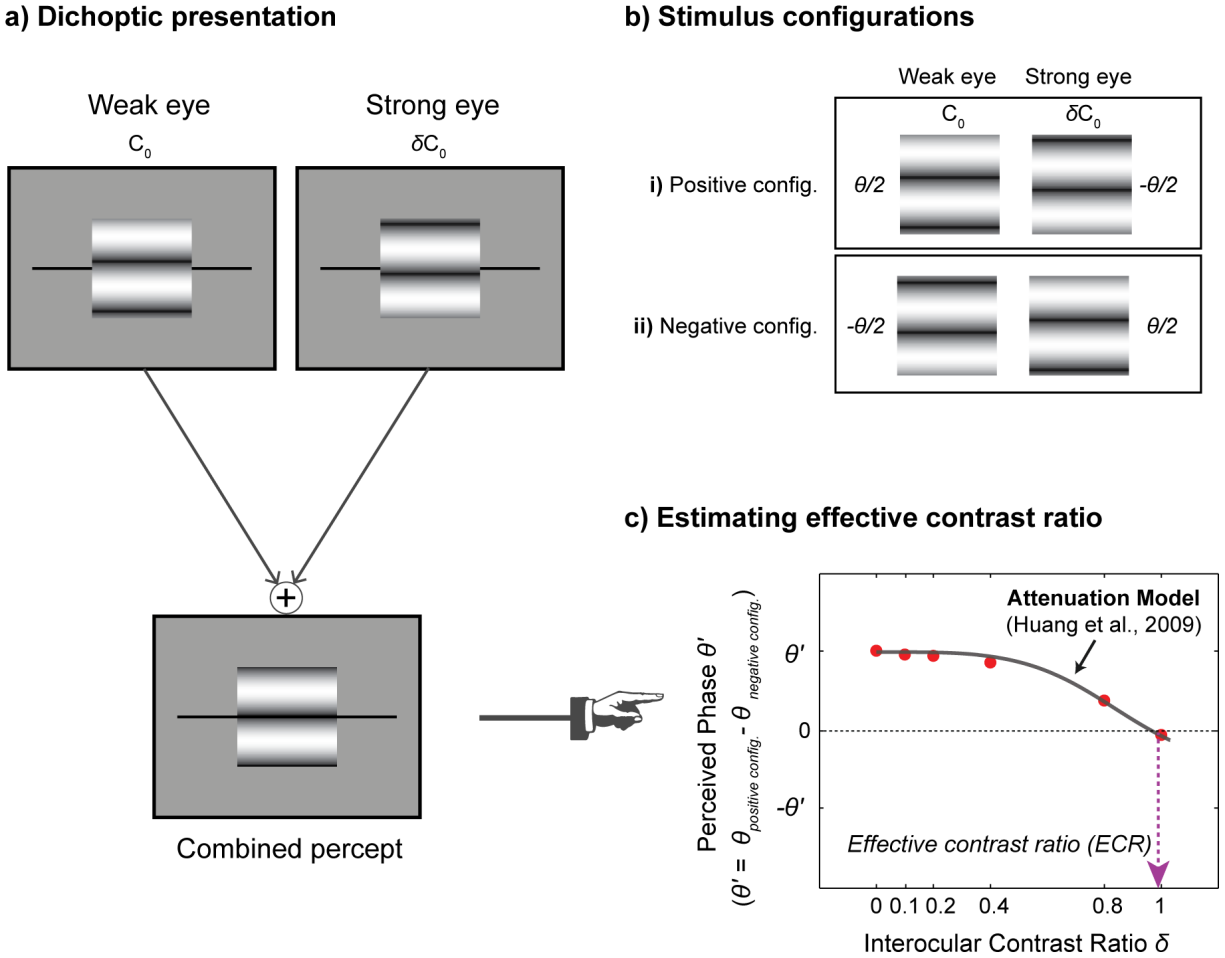
Schematic diagrams of stimulus configurations and task procedure. (**a**) Illustration of dichoptic stimulus presentation; (**b**) Illustration of two stimulus configurations. In the positive configuration, the phase of the grating in the weak eye was shifted by *θ/2* ( = 45°) from the midline while the phase in the strong eye was shifted by *-θ/2*. In the negative configuration, the phase-shift was reversed; (**c**) An example of the perceived phase (*θ*′) versus interocular contrast ratio *δ*. The perceived phase was measured as a function of interocular contrast ratio. The resulting data were fitted with the attenuation model [7] to compute effective contrast ratio. The black solid line is the best fit of the model. The dotted arrow line (magenta) indicates the estimated effective contrast ratio for this normally-sighted subject.



The test stimulus was a horizontal 1 cycle per degree (cpd) sinusoidal grating subtending 2°×2° placed in the center of a larger square (6°×6°, see Fig. 1a below). Stimuli were displayed on a uniform gray field at a viewing distance of 57*cm*. The stimulus contrast is expressed as Michelson contrast, which is defined as:
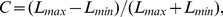
(1)where *L_max_* and *L_min_* are the maximum and minimum luminance of the stimulus respectively. The luminance profiles of the gratings to the weak and strong eyes are expressed as follows:
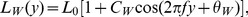
(2)


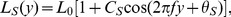
(3)where *L_0_* = 76 cd/m^2^ is the background luminance, *C_W_* and *C_S_* are the grating contrasts in the weak and strong eyes, *θ_W_* and *θ_S_* are the spatial phases of the gratings in the weak and strong eyes, and *f* = 1 cpd is the spatial frequency of the gratings. Each eye was shown exactly two cycles of the sine-wave gratings.

The stimuli were generated and controlled using MATLAB (version 7.9) with Psychophysics Toolbox extensions [39], [40] for Windows 7, running on a PC desktop computer (model: HP Pavilion). Stimuli were presented on a liquid crystal display monitor (model: ViewSonic VX2265wm; refresh rate: 120 Hz; resolution: 1680×1050) with the maximum brightness of 240 cd/m^2^. The monitor was calibrated using a spectrophotometer (model: Photo Research SpectraScan 655) and linearized. Subjects wore stereo-shutter glasses (nVidia Corp., Santa Clara, CA) running at 120 Hz frames rate.

### Procedure

As illustrated in Fig. 1a, two suprathreshold sine-wave gratings with the same spatial frequency (1cpd) were presented to the weak and strong eyes of the observer. A sine-wave grating was presented to one eye with phase shift *θ/2* above the midline and to the other eye with phase shift −*θ/2* below the midline, thereby producing a relative phase difference *θ* between the images in the two eyes that was fixed at 90°. The contrast of a sine-wave grating in the weak eye was fixed at 100% (*C_0_* = 1), the contrast in the strong eye was varied by the interocular contrast ratio *δ*. The perceived phase of the cyclopean sine-wave grating was measured with six interocular contrast ratios (*δ* = 0, 0.1, 0.2, 0.4, 0.8, 1).

The perceived phase *θ*′ of the cyclopean sine-wave grating was obtained with the method of adjustment. Subjects were instructed to indicate the apparent location of the dark stripe of the cyclopean image by moving a pair of black reference lines that bracketed the gratings (Fig. 1a). The reference lines were moved by the up- or down-arrow keys with a step size of 1 pixel (approximately 9° phase angle of the sine-wave grating). At the beginning of each trial, the reference line was randomly positioned relative to the center of the stimulus ([−9, 10] pixels). Subjects were asked to press the space bar on the keyboard when the lines were aligned with the center of the dark stripe to complete the trial. This was followed by a 500 ms inter-stimulus blank interval. The apparent location of the dark stripe of the grating defined the perceived phase of the cyclopean sine-wave grating. To minimize any potential up and down positional biases, two stimulus configurations were used (Fig. 1b): (i) *θ_W_* = −*θ_S_* = *θ*/2; (ii) *θ_W_* = −*θ_S_* = −*θ*/2. Measurements from configurations (i) and (ii) were then combined to build “perceived phase *θ*′ versus interocular contrast ratio *δ*″ functions (Fig. 1c).

The perceived phase *θ*′ was computed by subtracting the phase responses of the negative configuration from the phase responses of the positive configuration, adjusted for the random offset of the gratings, as shown in Fig. 1c. Thus, the maximum and minimum phases correspond to *θ* ( = 90°) and −*θ* respectively. Positive phase values indicate the cyclopean perception is dominated by the weak eye, while negative values indicate dominance by the strong eye. The interocular contrast ratio at which the perceived phase is zero indicates the contrast ratio at which the two eyes reached a balanced point. This balanced point is termed *effective contrast ratio*, and indicates the relative contrast required for the strong eye to match 100% contrast in the weak eye. Thus, the smaller effective contrast ratio, the more attenuated the input signal of the weak eye.

The order of stimulus configurations and interocular contrast ratios were randomized within a block of 36 trials (6 contrast ratios ×2 configurations ×3 repeats). All subjects completed at least one block of the task, which lasted approximately 8 mins. Subjects were given a few practice trials before the experimental test to make sure they fully understood the task and procedure. A chin-rest was used to maintain a constant viewing distance.

### Data analysis

To compute the effective contrast ratio of the weak eye, the perceived phase vs. interocular contrast ratio data were fitted with the attenuation model [7], [20]. The attenuation model can be expressed as the following equation:
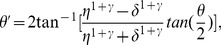
(4)where *θ* is the interocular phase difference, *δ* is the interocular contrast ratio, *γ* is the exponent of the power-law non-linearity in the contrast gain control process, and *η* is the effective contrast ratio. When the sinewave of one eye is absent (*δ* = 0), the perceived phase of the cyclopean grating is equal to that of the sinewave in the other eye (*θ*′* = θ*); when the effective contrasts of the two eyes are equal (*δ* = *η*), the perceived phase of the cyclopean image is equal to zero (*θ*′* = *0). *η* is defined as the effective contrast ratio of the weak eye. The model has two free parameters (see details of the model in [7], [20]). The model fit allows us to obtain the effective contrast ratio of the weak eye by estimating the interocular contrast ratio at which the perceived phase is 0° (i.e., the point where the two eyes' inputs reach equilibrium).

The curve fits were achieved using a simplex search method [41] to search for the optimal fit yielding the minimum least squares error. The fitting was performed on both individual subject's data and group average data. Representative data from one subject with normal vision are shown in Fig. 1c. The parameter values from the fit with group average data were consistent with the average parameter values from individual fits. The goodness-of-fit was assessed with the *r^2^* statistic [42]. The bootstrap resampling method [43] was used to estimate the mean and standard errors of the data for each subject group. This was done because we hoped to derive more stable and representative estimate of the population while minimizing any potential distortion from the relatively small samples (e.g., n = 6 for anisometric amblyopic group). The bootstrap procedure was performed with the following steps: i) construct 2000 resamples from the observed dataset (i.e., perceived phase vs. interocular contrast ratio) by sampling with replacement with a sample size of n; ii) compute the means and standard errors of re-sampled data; ii) fit the attenuation model to the bootstrapped group average data by using least squares method weighted by variance.

To examine if there were any significance differences in effective contrast ratio among subject groups and among different severity levels of stereoacuity and interoacular acuity difference (IAD), the data were analyzed using one-way analysis of variance (ANOVA) and Tukey's HSD pairwise comparison tests.

## Results

### Significantly lower effective contrast ratio for amblyopia

**Figure 2:**
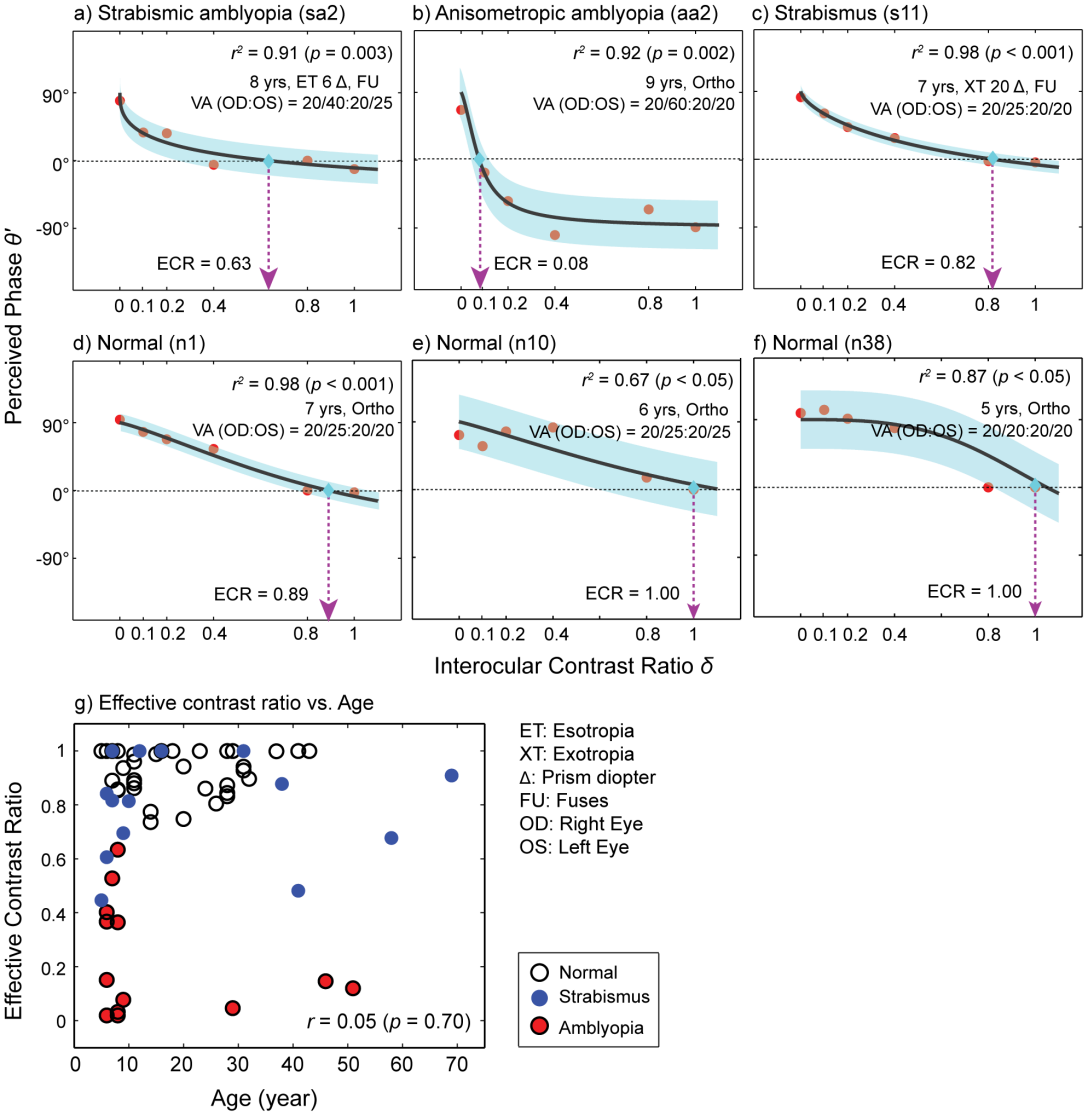
Examples of individual subject data. Each panel contains the perceived phase versus interocular contrast ratio function (red circles) of a representative subject from each group. Subject's age, angular eye deviation (type and amount of deviation), fixational information (fuses, left or right eye) and visual acuity are also shown in each panel. The data were fitted with the attenuation model (Eq. 4) to estimate effective contrast ratio (ECR) of the weak eye. The black solid lines are the best fits of the model. The dotted arrow lines (magenta color) indicate estimated effective contrast ratios. Shaded areas represent ±1 Standard Error of the Mean (SEM) of the fits. The goodness-of-fit was assessed with the *r^2^* statistic. (**a**) An individual with strabismic amblyopia (8 yrs, ET 6Δ, Fuses); (**b**) An individual with anisometropic amblyopia (9 yrs, ortho); (**c**) An individual with strabismus (7 yrs, XT 20Δ, Fuses); (**d**) A normally-sighted individual (7 yrs, ortho); (**e**) A normally-sighted individual (6 yrs, ortho); (**f**) A normally-sighted individual (5 yrs, ortho); (**g**) Correlation between effective contrast ratio and age (year). *ET: Esotropia, XT: Exotropia, Δ: Prism diopter, FU: Fuses, OD: Right eye, OS: Left eye. Note that the reported ocular deviation and fixational information are those made at near fixation (see Figs S1 and S2 for the data from all individual subjects).

Fig. 2 shows plots of the perceived cyclopean phase as a function of interocular contrast ratio. Each panel contains the data of a representative individual subject from each subject group (see Figs S1 and S2 for the data from all individual subjects). The effective contrast ratio (ECR) of the weak eye was estimated by fitting the data with the attenuation model (Eq. 4). Overall, the model fits were satisfactory with the mean *r^2^* values of 0.83 for strabismic amblyopia, 0.69 for anisometropic amblyopia, 0.84 for strabismus and 0.88 for normal (Table 2), indicating that about 69 to 88% of variance is accounted for by the model fit. The pattern of results summarized in Fig. 2 demonstrates that even after controlling for age, the effective contrast ratios of subjects with amblyopia were considerably lower than those of control subjects (i.e., strabismus without amblyopia and normal). Fig. 2g confirmed no significant correlation between effective contrast ratio and age (the Pearson product-moment correlation coefficient *r* = 0.05, *p* = 0.70), indicating that age did not contribute to the observed difference in effective contrast ratio among subject groups. As expected, the effective contrast ratios of normally sighted subjects were 0.89 (Fig. 2d), 1.0 (Fig. 2e) and 1.0 (Fig. 2f), indicating that the inputs from the two eyes contribute approximately equally to cyclopean perception. A similar, yet slightly lower contrast ratio (0.82) was observed in a subject with strabismus (Fig. 2c). On the other hand, substantially lower effective contrast ratios were observed in subjects with strabismic amblyopia (Fig. 2a) and anisometropic amblyopia (Fig. 2b), suggesting that the input from the weak eye was weighted much less than that of the strong eye in their suprathreshold cyclopean percept. This pattern of results was consistent with the findings of Huang et al.'s study [7]. They found that the effective contrast of the weak eye ranged from 0.11 to 0.28, indicating that only 11% to 28% of contrast is required for the strong eye to match 100% contrast in the weak eye.

**Table 2:** Mean effective contrast ratio (*η*), mean parameter value *γ* and mean *r* ^2^ value for the four subject groups.

	Strabismic amblyopia (N = 7)	Anisometric amblyopia (N = 6)	Strabismus (N = 15)	Normal (N = 40)
**ECR (** ***η)***	0.24 (±0.08)	0.20 (±0.09)	0.81 (±0.05)	0.93 (±0.01)
***γ***	5.90 (±5.25)	4.82 (±5.25)	3.00 (±1.18)	3.18 (±0.46)
***r^2^***	0.83 (±0.07)	0.69 (±0.08)	0.84 (±0.04)	0.88 (±0.02)

The number in parenthesis indicates ±1 Standard Errors of the Mean (SEM).

**Figure 3:**
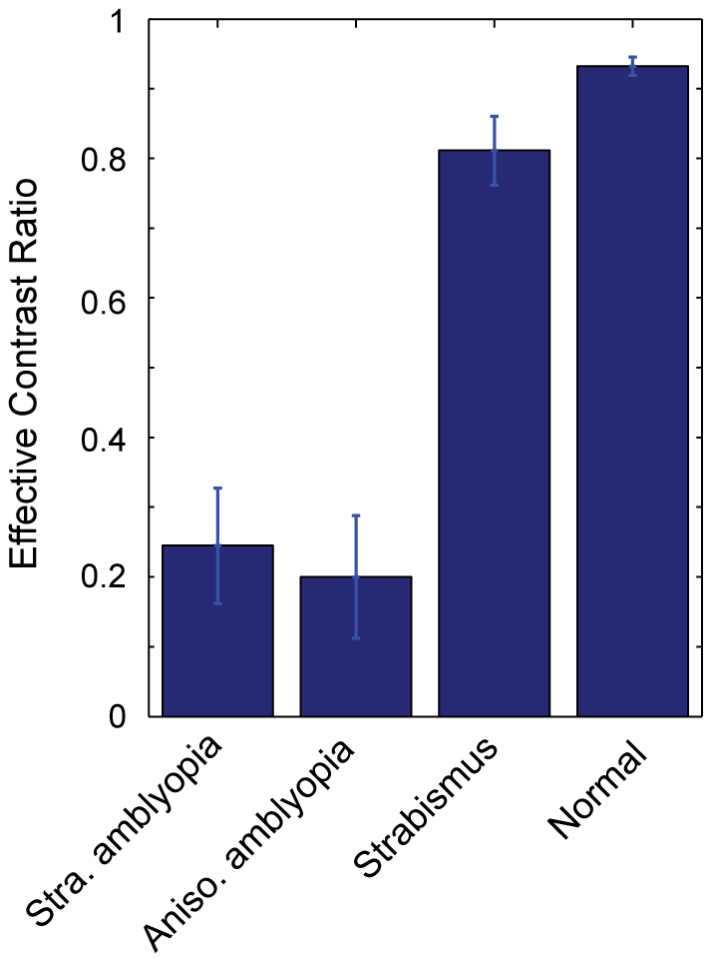
Mean effective contrast ratios for the four subject groups. Mean effective contrast ratio as a function of subject group. Error bars represent ±1 Standard Errors of the Mean (SEM).

**Figure 4:**
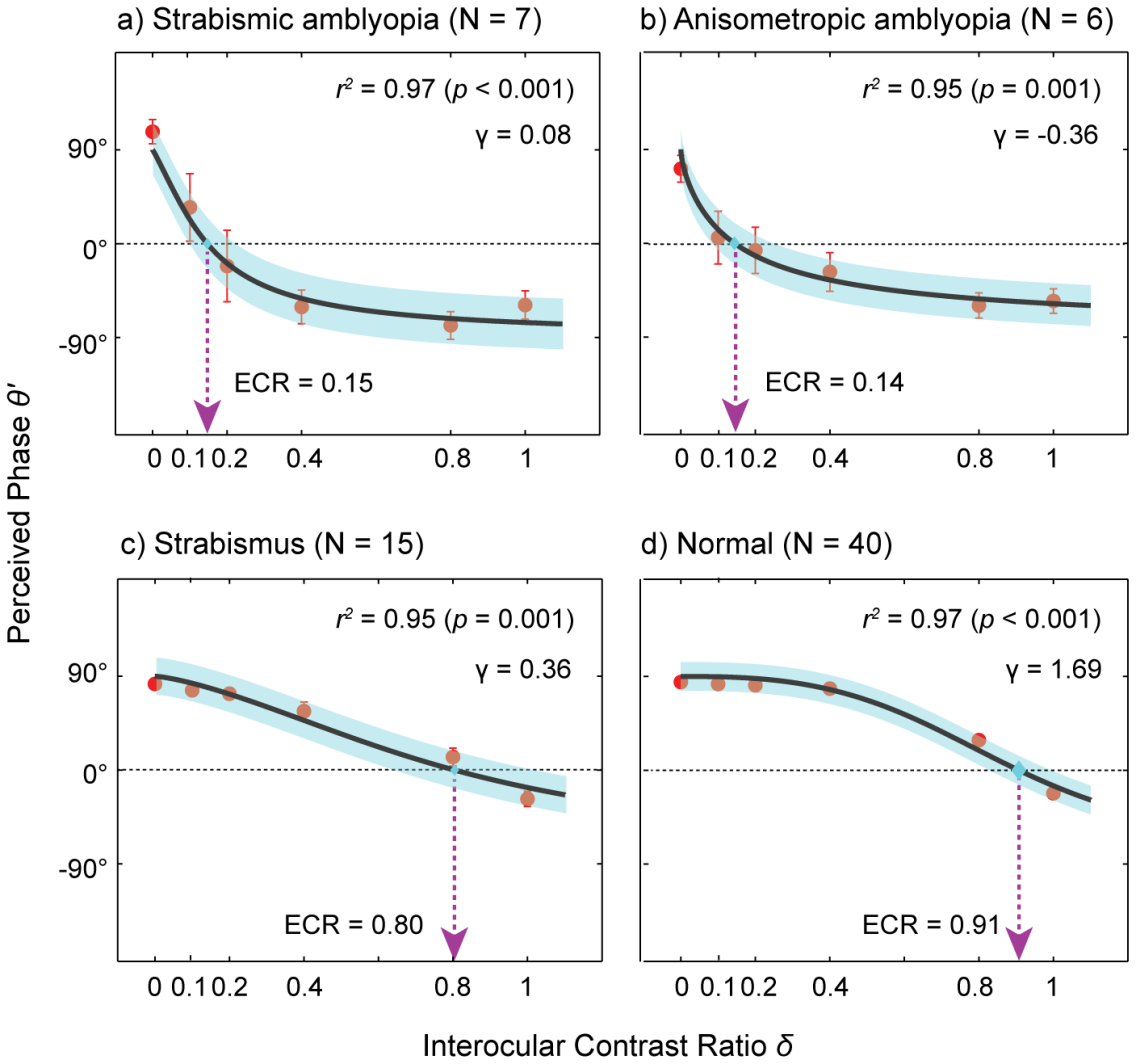
Group average data. Perceived phase plotted as a function of interocular contrast ratio for each subject group. The bootstrap resampling method [43] was performed to estimate the mean and standard errors of the data for each subject group. Each panel contains the average data (red circles) from each subject group. The average data were fitted with the attenuation model (Eq. 4) to obtain the effective contrast ratio of the weak eye. The black solid lines are the best fits of the model. The dotted arrow lines (magenta color) indicate estimated effective contrast ratios. Shaded areas represent ±1 SEM of the fits. The goodness-of-fit was evaluated by the *r^2^* statistic: (**a**) Strabismic amblyopia; (**b**) Anisometropic amblyopia; (**c**) Strabismus; (**d**) Normal.

Fig. 3 shows the mean effective contrast ratios for the four groups. A one-way ANOVA showed that there was a significant main effect of subject group (*F*_(3, 64)_ = 82.54, *p*<0.001) on effective contrast ratio. The ratios for strabismic amblyopia and anisometropic amblyopia were 0.24 (±0.083) and 0.20 (±0.09) while strabismus and normal vision showed 0.81 (±0.05) and 0.93 (±0.01). Tukey's HSD pairwise comparison test further revealed that the effective contrast ratios of the two amblyopic groups were significantly different from either strabismus or normal control (all *p*<0.001) while there was no significant difference between the two amblyopic groups. We also observed that the ratio of the strabismus without amblyopia group was significantly lower than that of the normal control group (*p*<0.05). Furthermore, this pattern of results is in good agreement with the outcome of the model fits to the group average data as shown in Fig. 4, suggesting that in both calculations, the pattern of results (i.e., ECR of amblyopia is significantly lower than those of controls) is similar even if the exact parameter values are not the same.

On the other hand, we did not find any significant difference in the parameter value of gamma (*γ*) between subject groups (*F*_(3, 64)_ = 0.48, *p* = 0.70). Table 2 summarizes mean effective contrast ratios (*η*), mean gamma values (*γ*) and mean *r* ^2^ values for the four subject groups.

### Relationships between effective contrast ratio and standard clinical measures of binocular visual function

**Figure 5:**
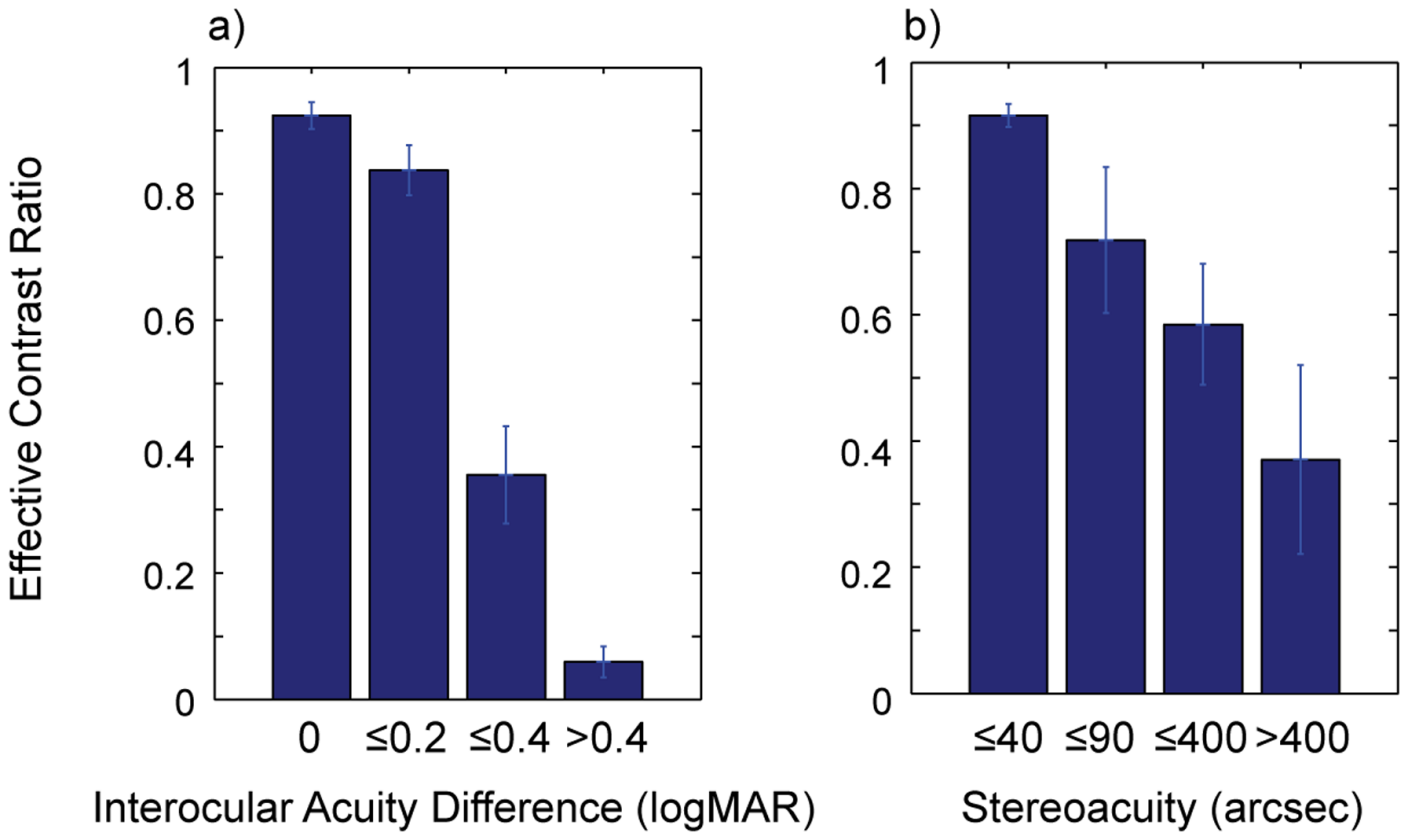
Relationships between effective contrast ratio and clinical binocular function measures. (**a**) Mean effective contrast ratios for four levels of IAD in logMAR units; (**b**) Mean effective contrast ratios for four levels of stereoacuity in acrsec units. Error bars represent ±1 SEM.

We then examined relationships between effective contrast ratio and clinical measures of binocular visual function such as stereoacuity and interocular acuity difference (IAD). Although IAD is not a direct measure of binocular visual function, we included IAD as an indicator of abnormal binocularity on the following grounds: IAD has been commonly used as one of the major criteria for diagnosing amblyopia and the linkage between large IAD and the loss of stereo-vision has been well established in previous studies [23], [44]–[46]. To perform the analysis, we first grouped subjects by the severity level of IAD and stereoacuity, and then computed the mean effective contrast ratio of each IAD and stereoacuity level. As shown in Fig. 5, there were close relationships between effective contrast ratio and the two clinical measures: effective contrast ratio decreased with increasing IAD and poor stereoacuity.

There was a significant main effect of IAD on effective contrast ratio (*F*_(3, 64)_ = 35.58, *p*<0.001). The effective contrast ratio decreased significantly with greater IAD (Fig. 5a). For example, the ratio decreased from 0.92 (±0.02) for the zero-IAD level to 0.06 (±0.02) for the IAD greater than 0.4 logMAR. Furthermore, Tukey's HSD pairwise comparison test showed that the contrast ratios of all four groups were significantly different from one another (all *p*<0.05) except for the 0-IAD and ≤0.2-IAD levels. Similarly, lower effective contrast ratio was associated with worse steroacuity (Fig. 5b). We found a significant main effect of stereoacuity on effective contrast ratio (*F*_(3, 64)_ = 13.45, *p*<0.001). The ratio reduced from 0.92 (±0.02) for stereoacuity less than 40 arcsec to 0.37 (±0.15) for stereoacuity greater than 400 arcsec. Tukey's HSD pairwise comparison test further showed that the effective contrast ratio of the ≤40-stereoacuity level was significantly different from either the ≤400-stereoacuity or >400-stereoacuity level (all p<0.01).

These findings demonstrated that our quantitative measure of the deficit in binocular interaction covaried with conventional clinical assessments of binocular function such as stereoacuity and IAD despite a great deal of inter-subject variability. These results are consistent with those of a previous study [23] showing that strong binocular imbalance measured by a dichoptic motion coherence task was associated with larger IAD and poorer stereoacuity.

### Test-retest reliability of the testing method

We next examined whether our test is stable over time. To this end, we invited a group of subjects (n = 10) including both normal vision and amblyopia back for a follow-up session and had them perform the same task again. Test-retest reliability was evaluated using i) Pearson's Product Moment Correlation coefficient (*r*) and ii) Bland & Altman difference plot [47] in which difference values between the two measurements (i.e., 1^st^ test-2^nd^ test) are plotted as a function of mean values of the two (i.e., (1^st^ test+2^nd^ test)/2) for each subject. A value of zero on the y-axis in a Bland & Altman plot indicates no change between two tests while larger deviation from the value of zero means larger variability between two tests. The mean difference value indicates any bias of the test while 95% limits of agreement mean that a difference value between the tests is likely to fall between the limits for most subjects (95%).

**Figure 6:**
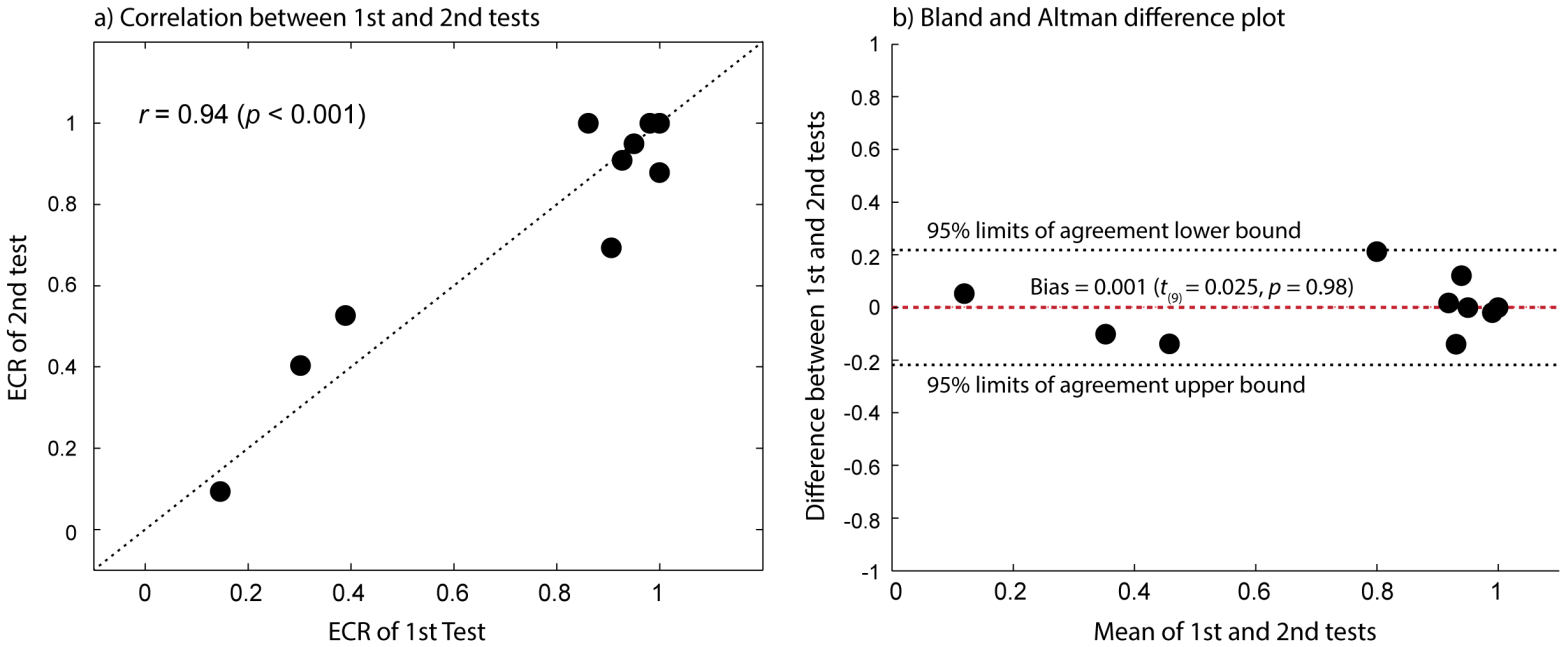
Test-retest reliability. (**a**) Correlation between 1^st^ and 2^nd^ tests. The dotted line indicates the line of equality (1^st^ test  = 2^nd^ test). Each black dot indicates a data point from each subject; (**b**) Difference in ECR between 1^st^ test and 2^nd^ test as a function of mean value of the two tests. Each black dot indicates a data point from each subject. The horizontal red dashed line represents a bias of the test, i.e., the mean difference value across subjects. The horizontal black dotted lines represent 95% limits of agreement.

Fig. 6a shows a plot of the ECR values of the 2^nd^ test against those of the 1^st^ test. The dotted line indicates the line of equality, where the ECR value of 1^st^ test is the same as that of 2^nd^ test across subjects. There was a good agreement between the two measurements indicated by Pearson's correlation coefficient (*r* = 0.94, *p*<0.001) while no noticeable bias was detected by the Bland & Altman difference plot (Fig. 6b). In other words, the observed mean difference value of 0.001 (±0.04) represented as the horizontal red dashed line in Fig. 6b was not statistically different from the value of zero (*t_(_*_9)_ = 0.025, *p* = 0.98).

## Discussion

Binocular vision can be described as the unification of two slightly different images transmitted from each eye to the visual cortex. At the cortical level, this visual information is fused into a single image and may be appreciated in three dimensions by the individual, known as stereopsis [48]. Binocular vision assists in the performance of various complex visual tasks, such as reading, object recognition, and visual-motor coordination [49], [50]. When normal binocular vision is disturbed by ocular misalignment (strabismus), unequal refractive errors (anisometropia), or any other condition causing unequal binocular inputs (e.g. cataract), the developing visual system is at risk of becoming amblyopic.

While abnormal binocular experience during early development is considered to be a major risk factor for amblyopia, current treatments deliberately suspend binocular vision. Penalizing the strong eye with patching, atropine, or optical blur, either completely eliminates input to one eye or provides a defocused image to the visual cortex. The focus of amblyopia treatment has been on improving the monocular function of the affected eye, and monocular visual acuity is the main outcome measure for amblyopia treatment. Penalizing the strong eye is an effective treatment that improves recognition and resolution visual acuity. However, many amblyopic individuals are left with residual deficits and are at risk for recurrent amblyopia. For example, approximately 15-50% of amblyopic children cannot achieve normal visual acuity and even when normal acuity is achieved, amblyopia often recurs [14].

There is compelling evidence that residual and recurrent amblyopia is likely attributed to remaining binocular dysfunction (see [14]). For instance, the risk of persistent amblyopia is more than 2 times greater among children with no stereoacuity compared to children with measurable stereoacuity [15]. Similarly, the risk of persistent amblyopia is more than 3 times greater for those with anisometropia of 1.00 D or more at the initial assessment compared with children with less than 1.00 D initial anisometropia [16]. These findings suggest a close linkage between binocular dysfunction and amblyopic deficits or perhaps, the severity of the amblyopic vision. In fact, many studies have already shown that the severity of amblyopic vision is closely related to interocular acuity difference and stereoacuity. For examples, Pardhan and Gilchrist [51] demonstrated that binocular summation decreases with induced interocular sensitivity difference, resulting in abnormal binocular vision. Goodwin and Romano [52] showed that reduction of stereoacuity is highly correlated with reduction of both monocular and binocular visual acuity in amblyopia.

To achieve more efficient diagnosis and disease management, it is imperative to be equipped with a reliable and rapid clinical assessment to quantify visual impairment thoroughly. In the past several years, various novel paradigms have been adopted to measure the binocular imbalance in amblyopia, such as the dichoptic global motion task [33], the dichoptic global orientation task [19], binocular phase combination task [7], [34], [35], and binocular phase plus contrast combination task [20], [36]. However, most of the studies have been conducted in laboratory settings requiring a relatively long testing time, which might not be practical for clinical use. In light of a need to transform the psychophysical assessments in a clinically viable format, Black et al. [37] are the first to develop a quick and compact version of test instrument for the binocular imbalance task and demonstrate its potential of implementing the test instrument in the clinic.

However, the questions arise whether the clinical assessment of binocular interaction can be carried out as a part of routine clinical assessments and whether the outcome of the assessment would produce meaningful information. The present study was therefore undertaken to demonstrate the feasibility of assessing binocular interaction in the clinic. In order to assess binocular interaction in amblyopia, we adopted a suprathreshold binocular summation paradigm developed by Ding & Sperling [34] which was successfully implemented to test binocular interaction in individuals with amblyopia by Huang et al.[7], [20] and by Ding et al. [35], [36]. In the current study, we transformed the original task into a quick dichoptic phase matching task, which is much faster and patient-friendly. In addition, we conducted the assessment in a local ophthalmology clinic during a patient's routine visit in order to evaluate the test in a typical clinical setting with a large number of naïve participants. Over a relatively short period of time (<8 mins), we were able to assess the interocular imbalance across a broad age range (5–69 years) in four different subject groups: anisometropic amblyopia, strabismic amblyopia, strabismus without amblyopia and normal.

We evaluated the efficacy of our method in three ways: First, to see if the outcome of our assessment is consistent with the findings from various interocular imbalance studies on amblyopia [7], [20], [23], [34], [35], [37], i.e., a considerable reduction in effective contrast in the amblyopic eye. Consistent with previous findings, we found that a significant difference in effective contrast between the fellow and amblyopic eye. More relevantly, our results confirmed the finding by Huang et al. [7] that the suprathreshold contrast signal in the weak eye of amblyopic observers was considerably attenuated relative to that in the strong eye. They found that the effective contrast ratios of the weak eye ranged from 0.11 to 0.28. The magnitude of this factor was greater than that predicted by interocular differences of contrast sensitivity or acuity. This result was consistent with our findings showing as low as 0.2 effective contrast for amblyopic individuals while non-amblyopic controls exhibited effective contrast of 0.8 to 0.9. We did not find any difference in effective contrast between anisometropic amblyopia and strabismic amblyopia. In view of previous findings of similar suprathreshold contrast perception in the amblyopic and fellow eyes [53], [54], a low ECR in amblyopia may seem surprising. Perhaps, this is because when suprathreshold stimuli are presented to both eyes to form a binocular percept, there may be an intrinsically inhibitory interaction from the fellow eye. This strong inhibitory interaction would not occur when suprathreshold contrast perception is probed monocularly. Thus, our findings together with those of previous studies [7], [20], [23], [34], [35], [37], show that normal or near to normal suprathreshold monocular perception does not necessarily guarantee normal binocular contrast summation.

Second, we further investigated the relationship between our binocular interaction measure and standard clinical measures indicating abnormal binocularity such as stereoacuity and interocular acuity difference (IAD). We found that our effective contrast ratio measure of binocular vision covaried with standard clincal measures of binocular vision such as IAD and stereoacuity. Despite a great deal of inter-subject variability, there was a significant decrease in effective contrast ratio with increasing amount of IAD and with degrading stereoacuity. These results are consistent with the findings of a previous study [23] showing that strong interocular imbalance measured by dichoptic motion coherence was associated with poorer binocular visual function including IAD and stereoacuity. This association between the two measures supports the validity of our measure in assessing binocular function. However, it is still unclear how and to what extent stereoacuity and IAD are related to the magnitude of interocular imbalance. There are various measures of binocular visual functions such as stereopsis, binocular summation, and interocular imbalance (e.g., ECR measure). While all provide an objective index of binocular visual function, each measure addresses apparently different aspects of binocular interactions. For instance, while stereoacuity likely taps into excitatory binocular interactions by evaluating the ability of the visual system to perceive depth from binocular disparity information, interocular imbalance likely taps into inhibitory interactions by determining the degree of unequal signal strength between the two eyes. For example, our data (Fig. 5) showed that ECR values varied across those individuals with the same IAD and stereoacuity, and vice versa, suggesting that different measurements reveal different aspects of binocularity.

While it has been long believed that amblyopic vision lacks excitatory binocular connections such as binocular summation, stereoacuity and interocular transfer [28]–[30], recent psychophysical studies have shown that binocular summation remains intact in some amblyopic individuals when the effective contrast of the two eyes are equated [7], [18], [21], [35], [36]. More importantly, studies on perceptual training have demonstrated that prolonged exposure to binocularly balanced stimuli can improve visual acuity and stereoacuity [24], [25]. We thus believe that timely and reliable assessment of interocular imbalance would help detect and monitor a deficit in binocular interaction, which cannot be captured by either visual acuity or stereoacuity.

It is noteworthy that a great deal of inter-subject variability has been observed in many amblyopia studies. For example, Vedamurthy et al. [55], [56] reported there was greater inter-subject variability in amblyopic individuals than normally sighted individuals in binocular interaction measured either by visual acuity, contrast detection or alignment sensitivity. Similarly, animal studies on the time course of visual recovery following early monocular deprivation showed a great deal of individual differences in the recovery of various visual functions [57], [58]. What might have caused these individual differences? Unfortunately, there is no simple answer to this question. We certainly cannot rule out a form of measurement and/or response errors and large individual differences in sensory and environmental factors that may occur among patient populations. Hence, our measurement of inter-subject variability may provide clinically meaningful information and help improve the detection, treatment and rehabilitation of the amblyopic vision through the development of customized therapies.

Third, we also examined the test-rest reliability of the test. Measurement is considered reliable when repeated measurements are stable over time. We found a high correlation between 1^st^ test and 2^nd^ test (*r* = 0.94, *p*<0.001) without any significant bias. This finding conveys confidence that our method can serve as a reliable method for assessing interocular imbalance.

Whether our findings from relatively low spatial frequency stimuli (1 cpd) can be generalized to other spatial frequencies requires further study. Relatively low spatial frequency gratings were used in the current study because judging the phase of a grating becomes harder and less accurate with increasing spatial frequency. Using low spatial frequency stimuli likely minimizes any potential confounds induced by spatial localization deficits that may occur at higher spatial frequencies [1], [59], [60]. While contrast sensitivity deficits at low spatial frequencies are less common in amblyopic vision [4], [54], [61], [62] compared to high spatial frequencies, evidence has suggested that normal monocular contrast sensitivity at a particular spatial frequency does not directly speak for normal binocular combination of that signal. For instance, Vedamurthy et al. [56] showed that while contrast sensitivity was normal at 4 cpd in the amblyopic eyes, binocular contrast sensitivity was still significantly lower than that expected from normal binocular summation, indicating abnormal binocular interaction at spatial frequencies with little or no measurable impairment for monocular contrast sensitivity. More relevantly, evidence has been accumulating that binocular contrast combination still remains abnormal even at 1 cpd or even 0.68 cpd. Previous studies [7], [20], [35], [36] using a similar suprathreshold binocular combination paradigm have demonstrated that the effective contrast ratio was significantly lower for amblyopia compared to normally-sighted individuals for either 1 cpd or 0.68 cpd spatial frequency. We believe that it is important to assess spatial-frequency dependent binocular interaction in amblyopia. Our subsequent study using stimuli less susceptible to spatial mislocalization and distortion indeed revealed that interocular imbalance becomes more pronounced with increasing spatial frequency in amblyopia. [63]

## Conclusions

Our results demonstrate that abnormal binocular interaction can be reliably captured by measuring the effective interocular contrast ratio. Our findings show that quantitative assessment of binocular interaction can be a quick, simple and repeatable test that quantifies the interocular imbalance in amblyopic vision, yet it is feasible to incorporate the assessment as a part of routine clinical assessments. We believe that reliable and timely assessment of deficits in a binocular interaction may improve detection and treatment of amblyopia.

## Supporting Information

Figure S1 **Individual subject data.** Each panel contains the perceived phase versus interocular contrast ratio function (red circles) of all subjects from each group. Subject's age, angular eye deviation (type and amount of deviation) and fixational information (fuses, left or right eye) are shown in each panel. The data were fitted with the attenuation model (Eq. 4) to estimate effective contrast ratio (ECR) of the weak eye. The blue solid lines are the best fits of the model. The dotted arrow lines (magenta color) indicate estimated effective contrast ratios. The goodness-of-fit was assessed with the *r^2^* statistic. (a) Individuals with strabismic amblyopia (sa); (b) Individuals with anisometropic amblyopia (aa); (c) Individuals with strabismus (s). *ET: Esotropia, XT: Exotropia, Δ: Prism diopter, FU: Fuses, OD: Right eye, OS: Left eye. Note that the reported ocular deviation and fixational information are those made at near fixation.
(TIF)

Figure S2 **Data from normally-sighted subjects (n).** The format of the plots is the same as Figure S1.
(TIF)
