# Supplementary figures and images for: Assessing Binocular Interaction in Amblyopia and Its Clinical Feasibility
Source: PLoS One. 2014 Jun 24;9(6):e100156. doi: 10.1371/journal.pone.0100156 (PMC4069064; doi:10.1371/journal.pone.0100156)

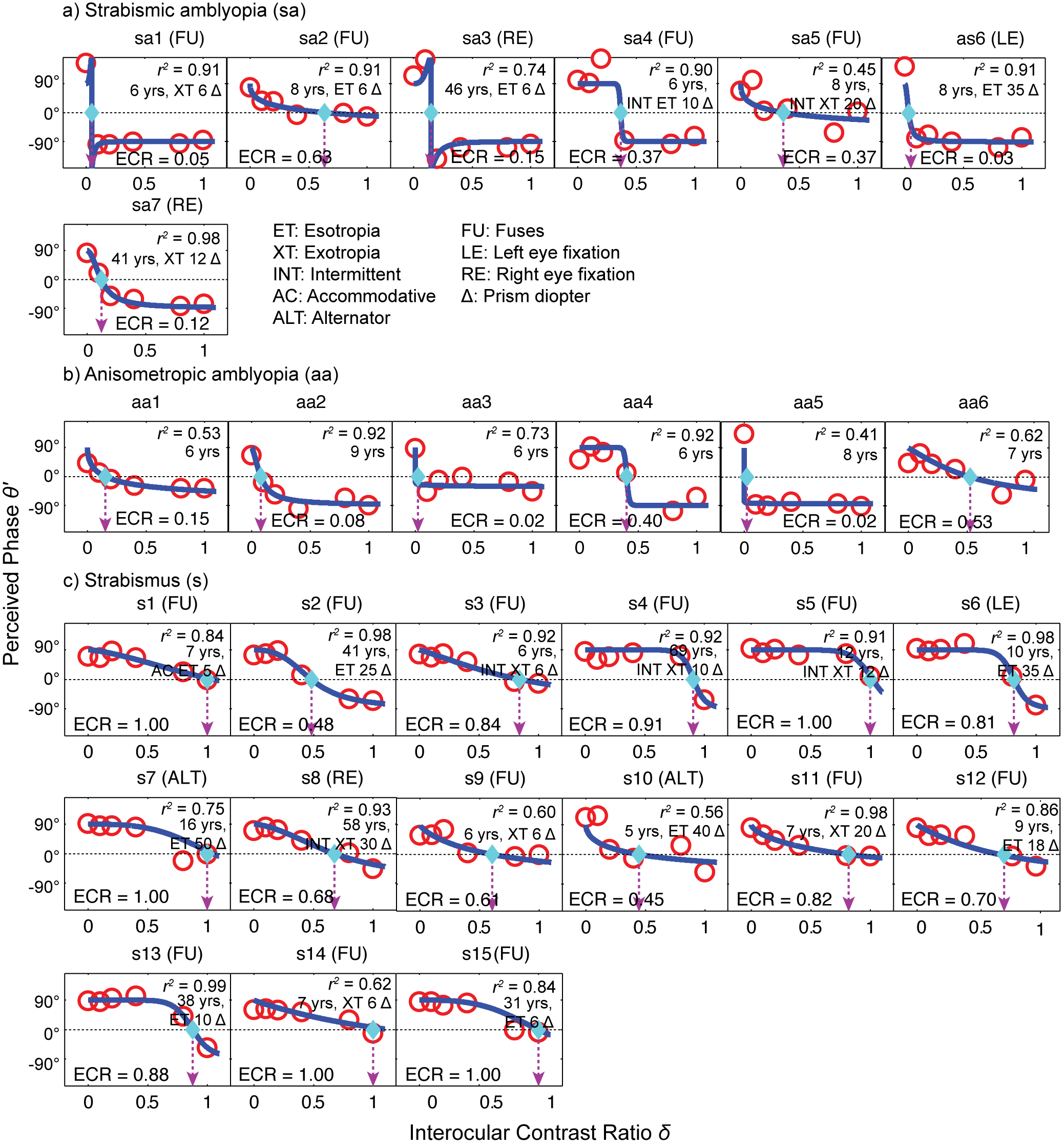

Supplement: Figure S1 — Individual subject data. Each panel contains the perceived phase versus interocular contrast ratio function (red circles) of all subjects from each group. Subject's age, angular eye deviation (type and amount of deviation) and fixational information (fuses, left or right eye) are shown in each panel. The data were fitted with the attenuation model (Eq. 4) to estimate effective contrast ratio (ECR) of the weak eye. The blue solid lines are the best fits of the model. The dotted arrow lines (magenta color) indicate estimated effective contrast ratios. The goodness-of-fit was assessed with the r2 statistic. (a) Individuals with strabismic amblyopia (sa); (b) Individuals with anisometropic amblyopia (aa); (c) Individuals with strabismus (s). *ET: Esotropia, XT: Exotropia, Δ: Prism diopter, FU: Fuses, OD: Right eye, OS: Left eye. Note that the reported ocular deviation and fixational information are those made at near fixation. (TIF) [file pone.0100156.s001.tif]

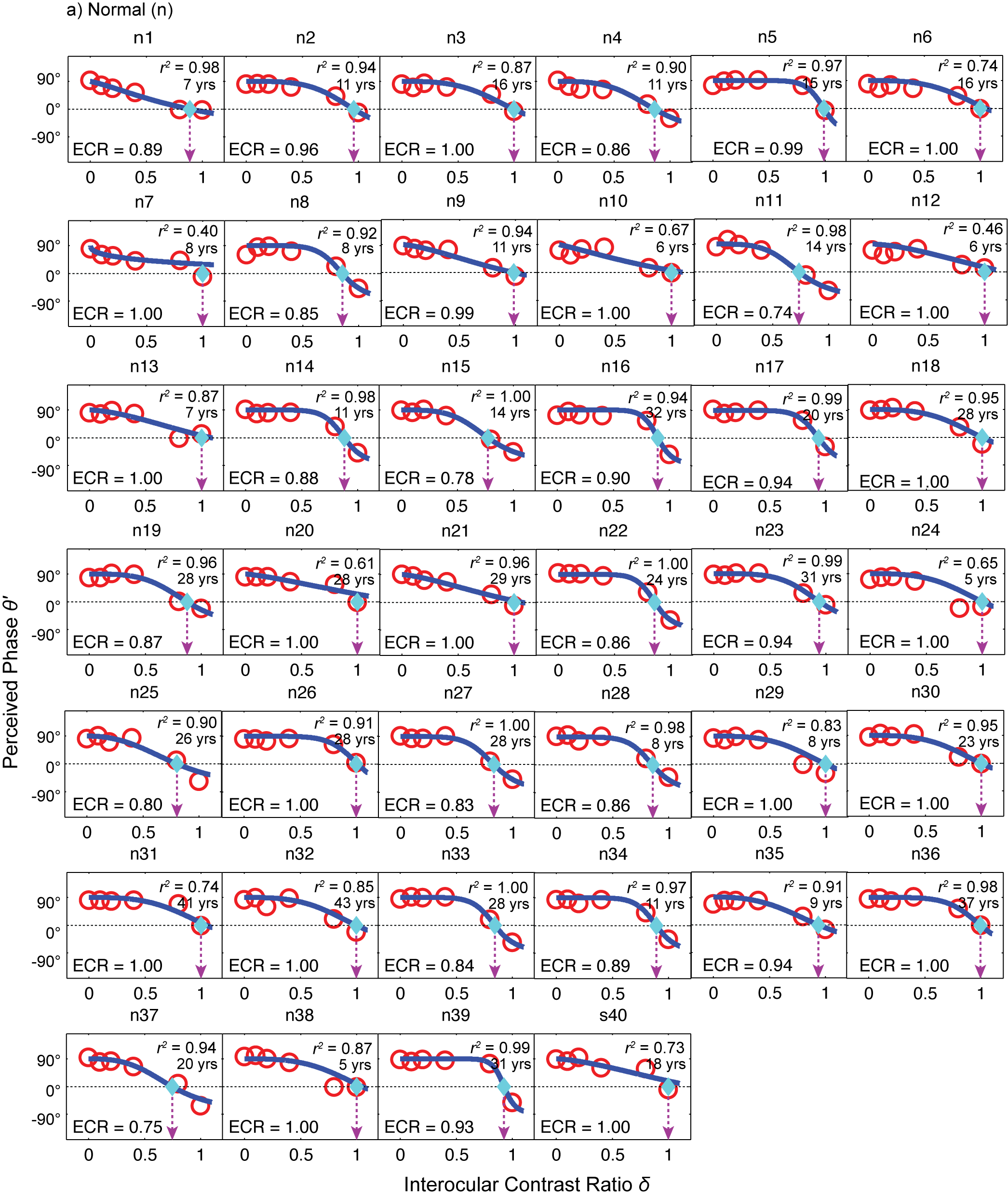

Supplement: Figure S2 — Data from normally-sighted subjects (n). The format of the plots is the same as Figure S1. (TIF) [file pone.0100156.s002.tif]
